# Quantitative assessment and genomic profiling of *Campylobacter* dynamics in poultry processing: a case study in the United Arab Emirates integrated abattoir system

**DOI:** 10.3389/fmicb.2024.1439424

**Published:** 2024-09-04

**Authors:** Ihab Habib, Mohamed-Yousif Ibrahim Mohamed, Glindya Bhagya Lakshmi, Hassan Mohamed Al Marzooqi, Hanan Sobhy Afifi, Mohamed Gamal Shehata, Mushtaq Khan, Akela Ghazawi, Afra Abdalla, Febin Anes

**Affiliations:** ^1^Veterinary Public Health Research Laboratory, Department of Veterinary Medicine, College of Agriculture and Veterinary Medicine, United Arab Emirates University, Al Ain, United Arab Emirates; ^2^ASPIRE Research Institute for Food Security in the Drylands (ARIFSID), United Arab Emirates University, Al Ain, United Arab Emirates; ^3^Food Research Section, Applied Research and Capacity Building Division, Agriculture and Food Safety Authority (ADAFSA), Abu Dhabi, United Arab Emirates; ^4^Food Technology Department, Arid Lands Cultivation Research Institute (ALCRI), City of Scientific Research and Technological Applications (SRTACITY), Alexandria, Egypt; ^5^Department of Medical Microbiology and Immunology, College of Medicine and Health Sciences, United Arab Emirates University, Al Ain, United Arab Emirates

**Keywords:** *Campylobacter*, chicken meat, enumeration, United Arab Emirates, whole genome sequencing, slaughterhouse

## Abstract

In the United Arab Emirates, no previous research has investigated the dynamics of the foodborne pathogen *Campylobacter* in broiler abattoir processing. This study conducted in one of the largest poultry producers in the UAE, following each key slaughter stage—defeathering, evisceration, and final chilling—five broiler carcasses were collected from 10 slaughter batches over a year. Additionally, one caecum was obtained from 15 chickens in each slaughter batch to evaluate the flock colonization. In total, 300 samples (150 carcasses and 150 caeca) were collected and enumerated for *Campylobacter* using standard methods. *Campylobacter* was pervasive in caecal samples from all slaughter batches, with 86% of carcasses post-defeathering and evisceration stages and 94% post-chilling tested positive for *Campylobacter*. *Campylobacter coli* predominates in 55.2% of positive samples, followed by *Campylobacter jejuni* in 21%, with both species co-existing in 23.8% of the samples. *Campylobacter* counts in caecal contents ranged from 6.7 to 8.5 log_10_ CFU/g, decreasing post-defeathering and evisceration to 3.5 log_10_ CFU/g of neck skin and further to 3.2 log_10_ CFU/g of neck skin post-evisceration. After chilling, 70% of carcasses exceeded 3 log_10_ CFU/g of neck skin. Whole-genome sequencing (WGS) of 48 isolates unveiled diverse sequence types and clusters, with isolates sharing the same clusters (less than 20 single nucleotide polymorphisms) between different farms, different flocks within the same farm, as well as in consecutive slaughter batches, indicating cross-contamination. Multiple antimicrobial resistance genes and mutations in *gyrA* T86I (conferring fluoroquinolone resistance) and an RNA mutation (23S r.2075; conferring macrolide resistance) were widespread, with variations between *C. coli* and *C. jejuni*. WGS results revealed that selected virulence genes (*pglG, pseD, pseI, flaA, flaB, cdtA,* and *cdtC*) were significantly present in *C. jejuni* compared to *C. coli* isolates. This study offers the first insights into *Campylobacter* dynamics in poultry processing in the UAE. This work provides a base for future research to explore additional contributors to *Campylobacter* contamination in primary production. In conclusion, effective *Campylobacter* management demands a comprehensive approach addressing potential contamination sources at every production and processing stage, guided by continued microbiological surveillance and genomic analysis to safeguard public health and food safety.

## 1 Introduction

*Campylobacter* spp. stands as the predominant foodborne bacterial pathogen behind human gastroenteritis globally, with broiler meat identified as a significant transmission source (Hansson et al., 2018; Habib et al., 2021). In the United States, contaminated broiler meat contributes to approximately 30% of foodborne campylobacteriosis cases, while in the European Union, an estimated 20–30% of campylobacteriosis instances are linked to the handling, preparation, and consumption of broiler meat (CDC, 2019; EFSA, 2020). Despite implementing rigorous general biosecurity measures at the farm level, preventing *Campylobacter* infection in broiler flocks towards the end of the rearing phase remains challenging (Pacholewicz et al., 2024). Additionally, despite efforts to minimize contamination during slaughter and processing, such as adherence to good manufacturing practices and stringent hygiene measures, carcass contamination remains a concern, particularly during evisceration, where intestinal rupture can occur (Zhang et al., 2018; Lassen et al., 2023).

Slaughter processing represents the final stage in the production process before chicken meat reaches consumers. Risk assessment studies suggest that the most effective short-term strategy for reducing human infections could be achieved by diminishing *Campylobacter* counts in contaminated slaughtered batches (Robyn et al., 2015; Foddai et al., 2022). Therefore, monitoring *Campylobacter* contamination and its dynamics throughout the slaughter processing is vital for pinpointing critical contamination points and preventing cross-contamination of chicken meat (Hakeem and Lu., 2021). Utilizing whole genome sequencing (WGS) could help provide in-depth insight into *Campylobacter* contamination in chicken abattoirs, facilitating precise identification of strains and transmission pathways (Foddai et al., 2022; Xiao et al., 2023). These advances in the sequence-based technique enable tracking of contamination sources and cross-contamination events with enhanced resolution, revealing genetic diversity, antimicrobial resistance, and virulence determinants, thus informing targeted intervention strategies to ensure safer poultry production (Foddai et al., 2022; Xiao et al., 2023).

The United Arab Emirates (UAE) is one of the top consumers of chicken meat in the Middle East, registering an annual consumption of approximately 50 kg *per capita* (USDA, 2021). In the UAE, the local poultry sector is primarily dominated by a small number (about six) of integrated businesses overseeing hatching facilities, farms, abattoirs, and distribution channels across the country (Habib et al., 2022). A previous retail-level study in the UAE found *Campylobacter* spp. present in 28.6% of fresh/chilled chicken carcasses, with 7% showing contamination levels of ≥3 log_10_ CFU/g (Habib et al., 2022). However, despite this considerable prevalence, there has been a notable absence of research focusing on *Campylobacter* at the abattoir or primary production levels in chicken slaughterhouses in the UAE. This gap presents a significant challenge for effectively monitoring and mitigating contamination risks, potentially compromising food safety standards and public health.

This study addresses this gap by presenting the first comprehensive examination of *Campylobacter* in the UAE abattoir setting. Employing a longitudinal approach, we sampled multiple abattoir batches over several months from a major integrated poultry production and processing establishment in the Emirate of Abu Dhabi in the UAE. Through detailed analysis of *Campylobacter* contamination dynamics and utilizing whole genome sequencing (WGS), we gained insights into genotypic diversity, antimicrobial resistance, and virulence determinants of *Campylobacter* clusters. This study enhances the understanding of *Campylobacter* epidemiology within UAE poultry processing and provides valuable data to the regional and global knowledge of this significant foodborne pathogen.

## 2 Materials and methods

### 2.1 Study site and sample collection

Samples were procured from a commercial slaughterhouse owned by a poultry establishment company in the Abu Dhabi Emirate of the UAE. This facility has the capacity to process 6,000 broiler carcasses per hour, employing the Halal slaughter method without stunning. The establishment maintains its own farms within a radius of fewer than 10 kilometers from the processing plant. Furthermore, the company contracts with various local farms to meet market demands, and provide them with one-day-old chicks, feed, and veterinary oversight. The company-owned slaughterhouse subsequently slaughters and processes the resulting flocks from these external farms. The company sources its one-day-old chicks from a broiler breeder in neighboring Oman. The water temperature in the scald tank ranges between 54°C and 55°C. Carcass chilling utilizes an air chilling system, with chiller air temperatures maintained between 2 and 5°C for 90 min.

A slaughter batch was defined as a group of birds from the same farm, raised in a broiler house during the same period (Seliwiorstow et al., 2015). During 10 visits from May to October 2023, we sampled 10 slaughter batches from seven flocks housed in internal farms and three flocks sourced from external farms. Five broiler carcasses were aseptically collected for each slaughter batch during each visit, from the following key slaughter step: defeathering, evisceration, and final chilling (Zhang et al., 2018). Additionally, one caecum was gathered from 15 chickens from each slaughter batch (Vinueza-Burgos et al., 2018). Initial samples were collected half an hour after the commencement of batch slaughter, with sample collection extending consecutively over 3 h (of which 1.5 h awaited to sample post-chill carcasses). All samples were carefully placed in sterile plastic bags, promptly cooled on-site using ice bricks, and transported to the laboratory under controlled cooling conditions. The slaughter process in this company predominantly occurs during the night, typically starting around 10:00 PM; hence, the samples were analyzed the subsequent day.

### 2.2 *Campylobacter* enumeration and identification

A total of 300 samples (comprising 150 carcasses and 150 caeca) were collected throughout this investigation. From the carcasses, approximately 10 grams of neck skin per carcass was incised using sterile scissors for the enumeration of *Campylobacter* (Seliwiorstow et al., 2015; Habib et al., 2019). Regarding the caeca (15 units per slaughter batch), each unit underwent immersion in ethanol, followed by the evaporation of ethanol, and subsequently, around 1 gram of content was squeezed into a sterile plastic cup, pooling the contents in an aseptic manner (Pacholewicz et al., 2015). Subsequently, all samples (both carcasses and caeca) were homogenized with 0.1% peptone water at a ratio of 1:10, followed by further serial dilutions (up to 10^−3^ for carcasses and 10^−6^ for caeca) and plating onto both mCCDA [modified charcoal cefoperazone deoxycholate agar (Neogen, United States)] and Rapid *Campylobacter* Agar (Bio-Rad, USA). These plates were then incubated under microaerobic conditions at 41.5°C for 48 h (ISO, 2017). After incubation, colonies exhibiting characteristic *Campylobacter* morphology were enumerated, with up to five colonies per sample being subjected to species-level confirmation utilizing Matrix-assisted laser desorption ionization-time of flight mass spectrometry (MALDI-TOF MS) methodology, employing the Autobio ms1000 instrument according to the manufacturer’s instructions (Autobio Diagnostics, China).

### 2.3 Whole-genome sequencing and computational analysis

For whole genome sequencing (WGS), a total of 50 isolates were chosen from seven slaughter batches that tested positive for *Campylobacter*. The samples were selected to reflect variability in farms (internal and external flocks) and in slaughter steps across positive batches. Genomic DNA extraction was performed using the Wizard^®^ Purification Kit (Promega, United States), followed by quality assessment with a Quantus fluorometer (Promega, United States).

Library preparation for WGS was performed using the Illumina NexTera XT kit (Illumina, United States) with 2×150 bp read length. Sequencing was performed using short-read technology (Illumina, NovaSeq) by a professional service provider (Novogene, the United Kingdom). The bioinformatics analysis was conducted using Solu, a cloud platform for real-time genomic pathogen surveillance (Moilanen et al., 2024). Solu integrates various tools and packages for analyzing WGS data, including BactInspector (Available at: https://gitlab.com/antunderwood/bactinspector) for species identification and MLST (Available at: https://github.com/tseemann/mlst) for determining multi-locus sequence types. AMR genes, and mutations, and virulence associated genes in bacterial genomes were annotated with AMRFinderPlus (Feldgarden et al., 2021), implemented via the Solu platform with default settings, where the threshold for gene identification was set at 90% (Moilanen et al., 2024). Additionally, A phylogenetic tree was constructed using the neighbor-joining algorithm, on the online platform SOLU (Solu Healthcare, Inc., Finland, https://platform.solu.bio/; accessed on 9th of May 2024) to visually represent evolutionary relationships among the isolates. The phylogeny results were computed using whole-genome SNPs, and is computed using a reference-free method (Available at “ska align”; https://github.com/simonrharris/SKA) that does not require a reference genome. This involved hierarchical grouping of similar genomes based on pairwise single-nucleotide polymorphism (SNP) distances, as elucidated by Fourment and Gibbs (2006). Clustering was defined by samples with ≤20 SNP pairwise distance (Hasan et al., 2021). Sequencing data generated in this study are available at the National Center for Biotechnology Information (BioProject no. PRJNA1112130).

### 2.4 Data analysis

The enumeration limit for *Campylobacter* was established at 10 colony-forming units per gram (CFU/g) for neck skin samples and 100 CFU/g for caeca samples (Rosenquist et al., 2013). When skin samples fell below the enumeration limit, quantification was adjusted to half of the enumeration threshold (Seliwiorstow et al., 2015). Before analysis, *Campylobacter* counts underwent a logarithmic transformation (log_10_) to approximate the data to normality for descriptive analysis (Habib et al., 2019).

Statistical analyses were conducted utilizing commercial software (Stata/MP 16, StataCorp LP, College Station, TX) with a significance level set at 5%. Differences in *Campylobacter* counts across sampling sites were assessed per batch employing the general linear model, specifically negative-binomial regression. Additionally, the degree of correlation between microbial counts was explored using linear regression for continuous data.

## 3 Results

### 3.1 *Campylobacter* status across slaughter batches

The findings presented in Table 1 reveal that *Campylobacter* colonization was pervasive across all examined slaughter batches, as indicated by positive detection in pooled caecal samples. Subsequent analysis showed that 86% of carcasses tested post-defeathering and evisceration stages, and 94% of carcasses sampled after final chilling were also positive for *Campylobacter* (Table 1). *Campylobacter* species were identified using the MALDI-TOF technique, revealing *Campylobacter coli* as the predominant species in 55.2% of the positive samples from various sampling sites, followed by *Campylobacter jejuni* in 21% of cases. Notably, both species co-existed in 23.8% of the positive samples.

**Table 1:** Campylobacter detection across processing steps of different slaughter batches.

Slaughter batch	Campylobacter status across processing stages (+ve / total sampled)			
	Polled caeca (*n* = 15 per batch)	After defeathering	After evisceration	After final chilling
1	*Campylobacter* + ve	5/5	5/5	5/5
2	*Campylobacter* + ve	5/5	5/5	4/5
3	*Campylobacter* + ve	5/5	5/5	5/5
4	*Campylobacter* + ve	4/5	5/5	5/5
5	*Campylobacter* + ve	5/5	5/5	5/5
6	*Campylobacter* + ve	5/5	5/5	5/5
7	*Campylobacter* + ve	2/5	0/5	3/5
8	*Campylobacter* + ve	3/5	3/5	5/5
9	*Campylobacter* + ve	5/5	5/5	5/5
10	*Campylobacter* + ve	4/5	5/5	5/5
Total		86% (43/50)	86% (43/50)	94% (47/50)

### 3.2 Dynamics of *Campylobacter* counts across processing steps

**Figure 1:**
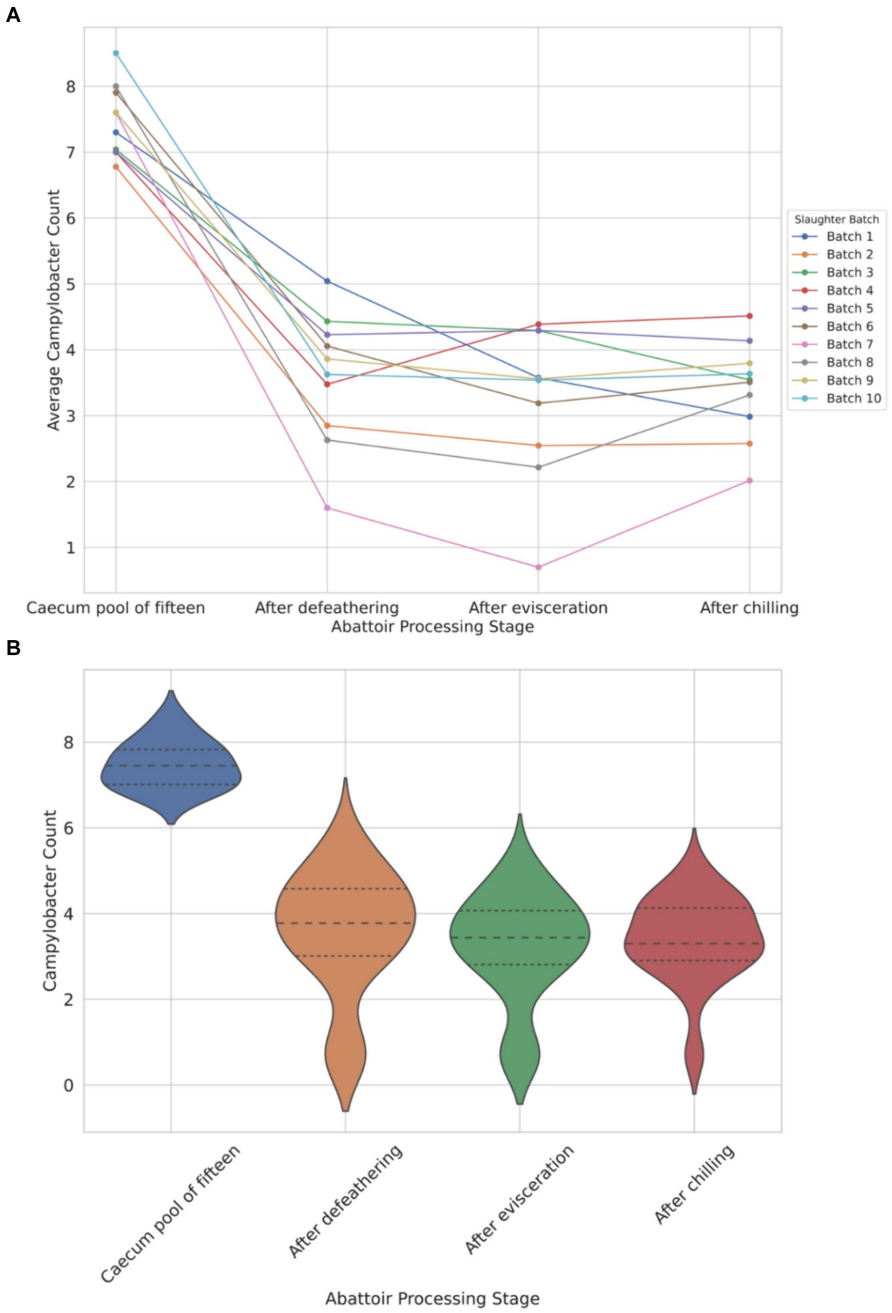
Comparison of *Campylobacter* counts (log_10_ CFU/g) presented individually across each of the 10 slaughter batches **(A)** and violin plot view of the distribution of counts across four different abattoir processing stages **(B)**.

The data depicted in Figure 1A illustrates a notable load of *Campylobacter* colonization in caecal contents across slaughter batches, ranging from a minimum of 6.7 log_10_ CFU/g to 8.5 log_10_ CFU/g. Following the defeathering step, the average *Campylobacter* count on carcasses was 3.5 log_10_ CFU/g (with a standard deviation (SD) of ±1.4 log_10_ CFU/g), while after evisceration, the average count decreased slightly to 3.2 log_10_ CFU/g (SD ±1.2 log_10_ CFU/g). Figure 1B shows that carcass samples taken post-chilling had an average *Campylobacter* count of 3.4 log_10_ CFU/g, with the narrowest distribution (SD of ±0.9 log_10_ CFU/g), indicating a more consistent reduction in *Campylobacter* counts after chilling.

The violin plot (Figure 1B) visually represents the overall trend, demonstrating reduction and stabilization as processing progresses from the caecum pool to the chilling stage. Chicken carcasses with *Campylobacter* counts surpassing 3 log_10_ (1,000) CFU/g constituted 74% after defeathering, 62% after evisceration, and 70% after chilling (Figure 1B).

**Figure 2:**
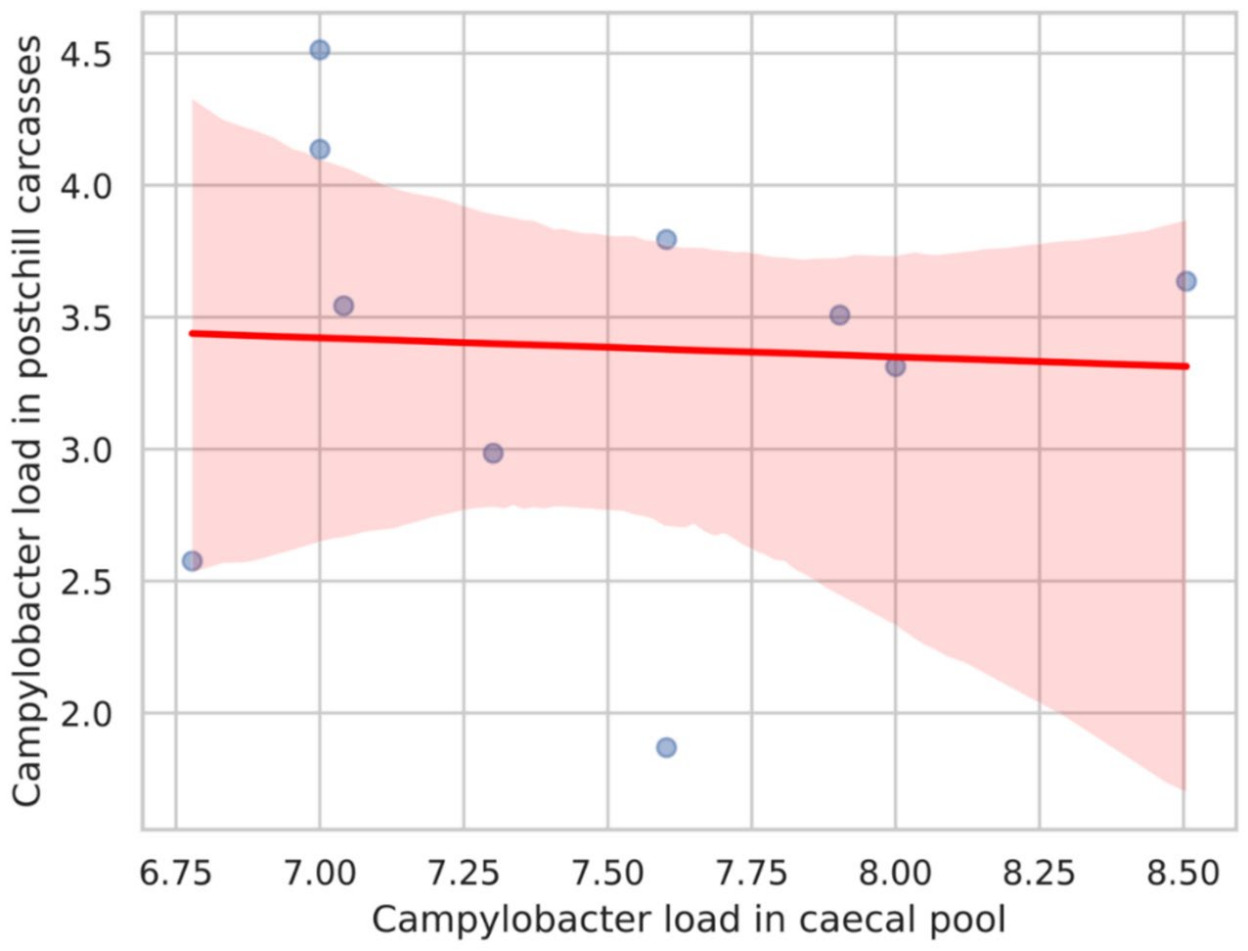
Scatter plot with a regression line showing the correlation between the *Campylobacter* load in the caecal pool and the *Campylobacter* load in postchill carcasses.

Figure 2 presents the Pearson correlation coefficient between *Campylobacter* counts in the caecal pool and post-chill carcasses, which was calculated as −0.052. This value suggests a very weak negative correlation between the two variables. Consequently, the weak correlation implies that the *Campylobacter* load in the caecal pool does not strongly predict the *Campylobacter* counts in post-chill carcasses.

### 3.3 Clusters and diversity based on WGS analysis

**Figure 3:**
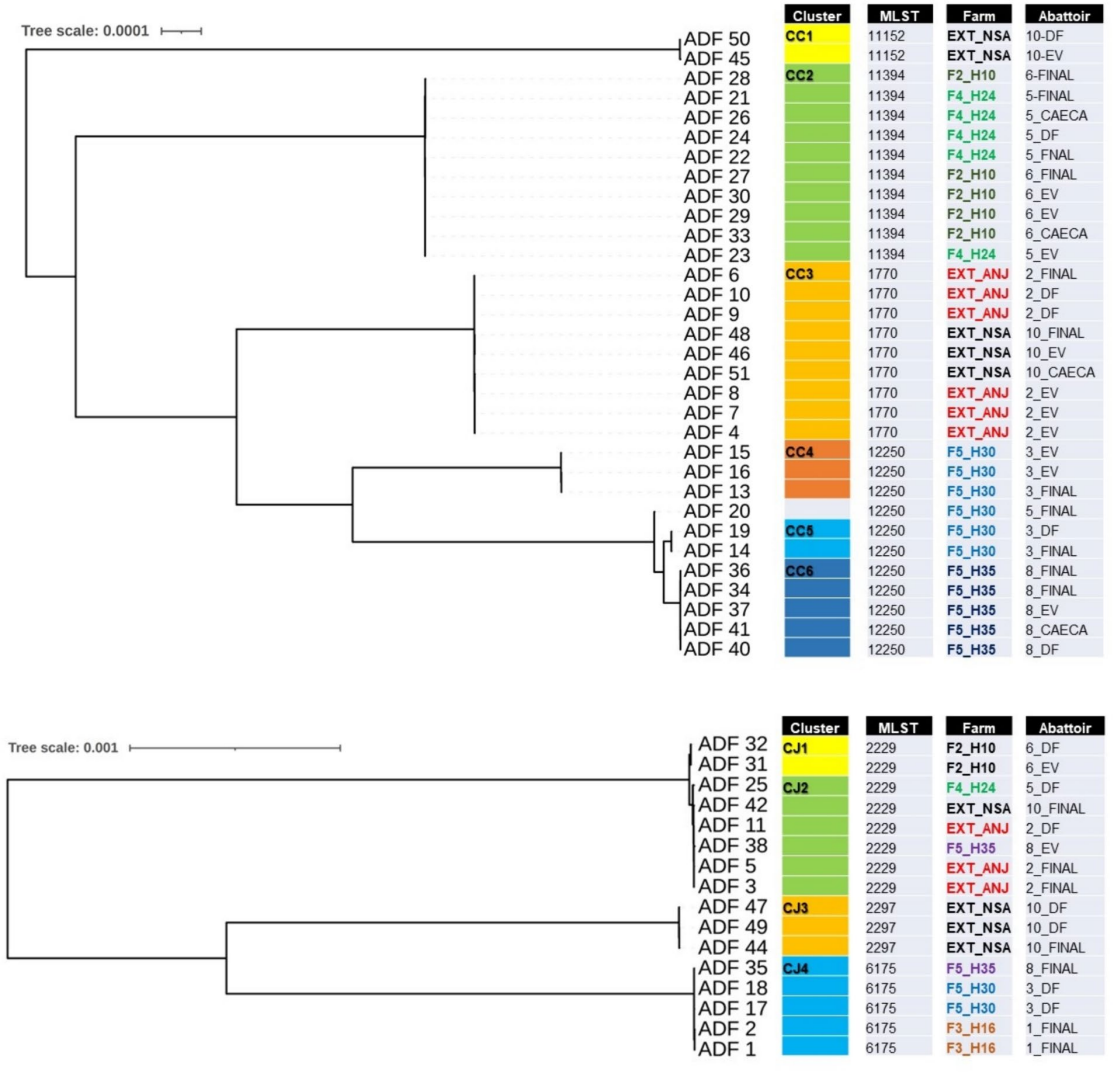
Single-nucleotide polymorphism (SNP) phylogenomics tree of 32 whole-genome sequenced *Campylobacter coli* **(A)** and 16 *Campylobacter jejuni* **(B)** isolates recovered from chicken carcasses sampled from different farms and abattoir processing stages (DF, defeathering; EV, evisceration; FINAL, after final chilling; CAECA, pooled caeca). Any two samples that have less than 20 SNP distance will be considered a cluster.

Of the 50 isolates selected for further WGS, two were found to be contaminated with mixed species and were consequently excluded from the subsequent analysis. Figure 3 illustrates two phylogenomics trees, one for *Campylobacter coli* (*n* = 32) isolates and the other for *Campylobacter jejuni* (*n* = 16) isolates. Among the *C. coli* isolates, four multilocus sequence types (STs) and six clusters (CC1 to CC6) were identified (Figure 3A). CC1 (ST-11152) and CC3 (ST-1770) were associated with isolates recovered from externally contracted farms. CC2 (ST-11394) included isolates from two different slaughter batches originating from separate internal farms (F4_H24 and F2H10), indicating a potential cross-contamination between these farms. Moreover, CC4, CC5, and CC6 all had the same sequence type (ST-12250) and contained isolates from the same farm but different flock houses (F5_H30 and F5_H35), which suggests that contamination could have spread within the farm and then to slaughter batches. The detailed single nucleotide polymorphism (SNP) distance matrix among *C. coli* isolates is available in Supplementary Table S1.

For *C. jejuni*, SNP phylogeny revealed four clusters (CJ1 to CJ4), with CJ1 and CJ2 aligned to ST-2229 (Figure 3B). However, CJ1 originated from a unique internal farm and slaughter batch, whereas cluster CJ2 included isolates from both external and internal farms, appearing in slaughter batches 2, 5, and 10. This observation potentially indicates a cluster of cross-contaminated isolates, that likely persisted in the abattoir environment. The detailed SNP distance matrix among *C. jejuni* isolates is presented in Supplementary Table S2.

### 3.4 Genome insight on antimicrobial resistance and virulence factors

**Figure 4:**
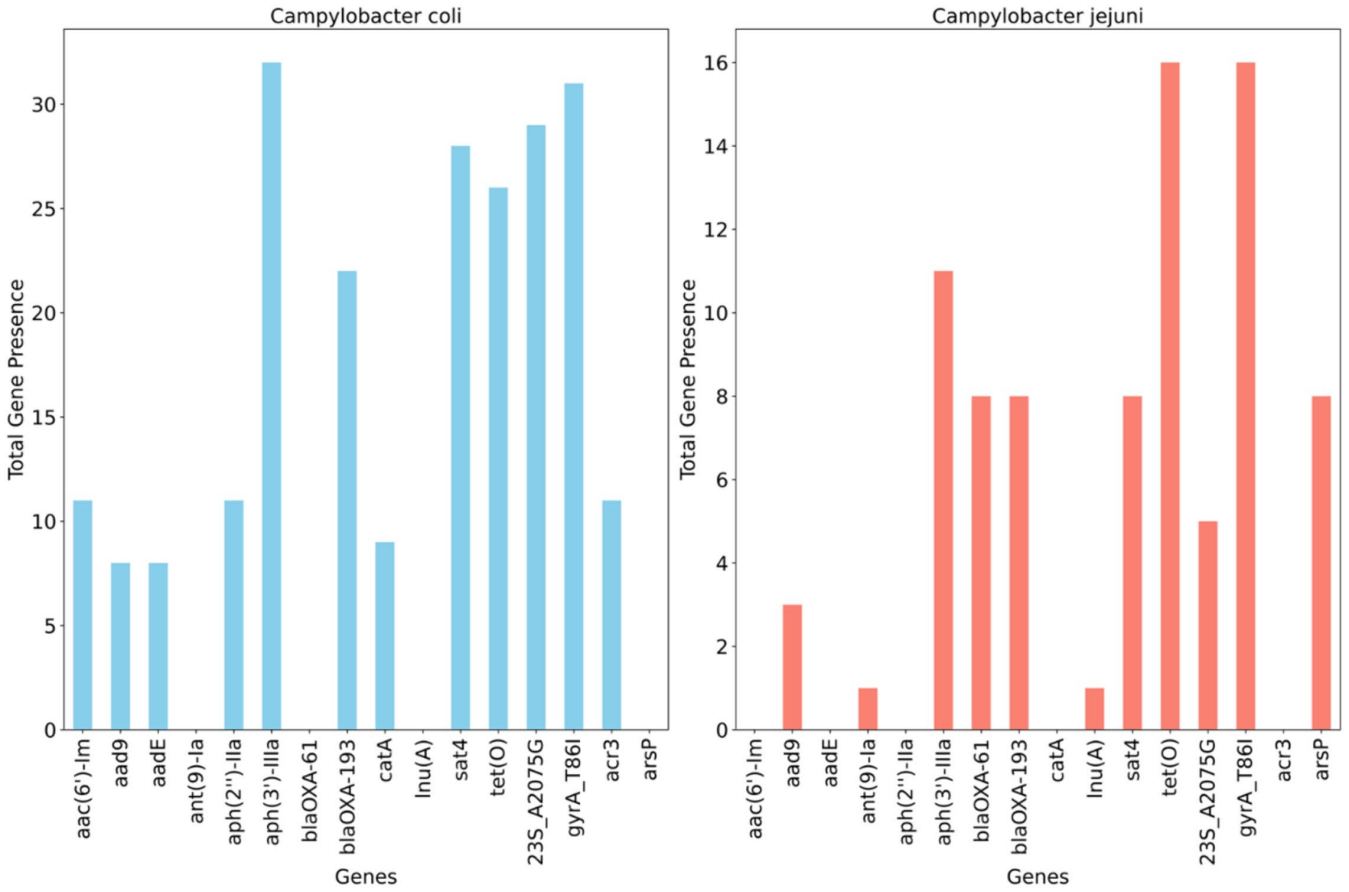
Distribution of antimicrobial resistance genes and mutations across whole genome sequenced *Campylobacter coli* (*n* = 32) and *Campylobacter jejuni* (*n* = 16) detected in chicken carcasses sampled from different farms and abattoir processing stages.

Figure 4 illustrates the detection of multiple antimicrobial resistance genes in *C. coli* and *C. jejuni*, with 10 and 9 genes identified, respectively. Several aminoglycoside resistance genes were prevalent among the isolates, with *aph(3′)- IIIa* being the most frequently encountered gene, found in 100% (32/32) of *C. coli* and 68.7% (11/16) of *C. jejuni* isolates. The streptomycin resistance gene (*aadE*) was present in 25% of *C. coli* isolates but absent in *C. jejuni*. Additionally, the *bla*_OXA–61_ gene, conferring resistance to β-lactamases, was observed in 50% (*n* = 8) of *C. jejuni* isolates but not in *C. coli*, whereas *bla*_OXA–193_, another ampicillin resistance gene, was found in 58.3% (*n* = 21) of *C. coli* and 50% (*n* = 8) of *C. jejuni* isolates. The *catA* gene, associated with phenicol resistance, was detected in 25% (*n* = 8) of *C. coli* but was absent in *C. jejuni*. Conversely, the methylarsenite efflux permease (*ArsP* gene), conferring resistance to organoarsenicals, was present in 50% (8/16) of *C. jejuni* isolates but not in *C. coli* (Figure 4).

A single point mutation (T86I) in the housekeeping gene *gyrA*, conferring resistance to (fluoro) quinolones, was identified in 96.8% of *C. coli* and all *C. jejuni* isolates. An RNA mutation (23S r.2075), associated with macrolide resistance (nucleotide change, A - > G), was present in 84.3% (27/32) of characterized *C. coli* isolates compared to 31.2% (5/16) of *C. jejuni* isolates (Figure 4).

**Figure 5:**
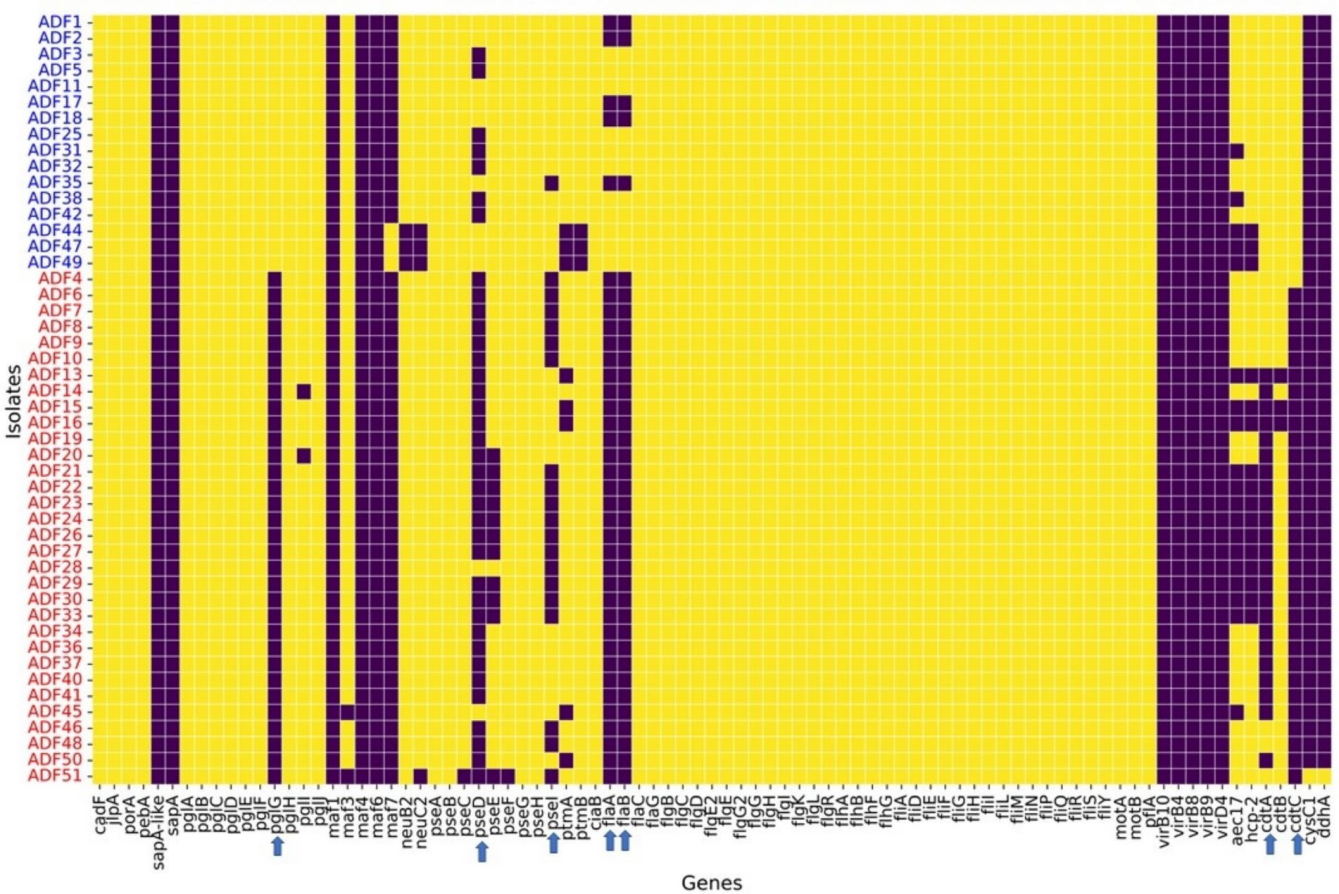
Virulence factors found in whole-genome sequenced *Campylobacter coli* (red coded isolated) and *Campylobacter jejuni* (blue coded isolates) recovered from chicken carcasses sampled from different farms and abattoir processing stages. The heatmap visualizes the presence or absence of specific genes across different isolates grouped by *Campylobacter* species, as indicated by the sample codes on the Y-axis. Each row represents an isolate, and each column corresponds to a specific gene. The color in the heatmap indicates whether a gene is present (yellow color) or absent (blue color) in an isolate.

Regarding virulence factors (Figure 5), WGS revealed notable variation between *C. coli* and *C. jejuni* isolates in the distribution of virulence factors. Genes associated with O-linked protein glycosylation (*pglG*), biosynthesis of pseudaminic acid (*pseD* and *pseI*), flagellin structural genes (*flaA* and *flaB*), and genes encoding the cytolethal distending toxin (*cdtA* and *cdtC*) were more prevalent in *C. jejuni* compared to *C. coli* (Figure 5). These differences were statistically significant (*p*-value <0.05), as determined by logistic regression analysis. Moreover, plasmid-mediated virulence genes, including *virB*-D encoding the Type IV secretion system, along with other *virB* gene clusters, were absent in the genomes of all isolates. Additionally, several genes of the motility accessory factor (*maf*) family, related to flagellar biosynthesis and phase variation, were also absent from all isolates.

## 4 Discussion

*Campylobacter* infection is the leading cause of bacterial gastroenteritis in both developed and developing countries (CDC, 2019; EFSA, 2020). Despite the United Arab Emirates (UAE) ranking among the prominent consumer markets for chicken meat, limited data on *Campylobacter* exists, particularly during broiler production. To the best of our knowledge, no previous research in the UAE has assessed *Campylobacter* dynamics and prevalence during abattoir processing, underscoring the significance of the present study. Focusing on one of the major producers of fresh chicken meat in the UAE, we examined the contamination rate of *Campylobacter* in samples collected across the slaughter line, ranging from the caecum in freshly slaughtered birds to post-chill carcasses.

In the present study, *Campylobacter* counts exceeding 6.5 log_10_ CFU/g were detected in pooled caecal content samples, consistent with findings from previous research (Allen et al., 2007; Vinueza-Burgos et al., 2018). Following defeathering, the mean *Campylobacter* count on neck skin across various slaughter batches was 3.5 log_10_ CFU/g, slightly elevated compared to findings in other industrial slaughterhouse studies (Seliwiorstow et al., 2015; Vinueza-Burgos et al., 2018). While evisceration is traditionally identified as a critical stage for *Campylobacter* contamination of carcasses, inconsistent increases in *Campylobacter* counts during this step have been reported by other researchers (Pacholewicz et al., 2015; Lassen et al., 2023). In our investigation, the average *Campylobacter* count post-evisceration (3.2 log_10_ CFU/g on neck skin) was comparable to the average counts after defeathering. This finding possibly suggests that intestinal leakage during evisceration in our study site may have minimal impact on *Campylobacter* contamination. Furthermore, we found that the distribution of *Campylobacter* counts becomes narrower post-evisceration, indicating a potential role of efficient evisceration practices in consistently reducing counts compared to earlier stages across the processing line (Keener et al., 2004). After chilling, we observed the most constrained distribution of *Campylobacter* counts on carcasses tested in this study, indicating a desirable uniform reduction in counts. The air chilling protocol employed in this facility (90 min at 4°C to 5°C) appears to be a critical contributor to diminishing and stabilizing *Campylobacter* counts across diverse slaughter batches (Leone et al., 2024).

The weak correlation coefficient observed between *Campylobacter* counts in the caecal content and post-chill carcasses suggests the influence of additional factors on post-chill *Campylobacter* levels, a phenomenon also noted in previous studies elsewhere (Seliwiorstow et al., 2015; Foddai et al., 2022). These factors may encompass variations in processing methods, effectiveness of chilling systems, risks of cross-contamination, and differences in handling and sanitation protocols (Nagel et al., 2013; Pacholewicz et al., 2015; Leone et al., 2024). Thus, addressing *Campylobacter* contamination at the current study site requires a comprehensive approach that considers multiple factors beyond reducing only the initial bacterial load in the caecal pool. Notably, our study revealed that 70% of post-chill carcasses were contaminated with more than 1,000 CFU of *Campylobacter* per gram of neck skin, likely due to the origin of all slaughter batches from *Campylobacter*-colonized flocks from both internal and external farms to the poultry establishment. Given the established association between highly contaminated broiler carcasses and the risk of human *Campylobacter* infection (Duarte and Nauta, 2015), the prevalence of such highly contaminated carcasses in the present poultry establishment is concerning.

The analysis of 48 genomes in this study revealed three sequence types within *C. jejuni* and four within *C. coli*. Notably, *C. coli* ST-12250 was previously unrecognized and identified for the first time exclusively in the UAE, and we reported it before in *Campylobacter* samples from retail carcasses across four other companies (brands) (Habib et al., 2023). This highlights the importance of regional variations in *Campylobacter* sequence types, with the unique identification of *C. coli* ST-12250 in the UAE extending its presence across diverse local poultry businesses. Furthermore, examination of the *Campylobacter* PubMLST database [https://pubmlst.org/organisms/campylobacter-jejunicoli, (accessed May 13, 2024)] indicates that the majority of sequence types identified in this study have been documented in other countries, across Europe and in the United States, and have been associated with both chicken and human sources. This underscores the global distribution and interconnectedness of *Campylobacter* sequence types and the potential for zoonotic transmission of the identified sequence types in both *C. coli* and *C. jejuni* originating from the investigated poultry establishment.

This study showcases the potential of whole-genome sequencing (WGS) as a vital tool for elucidating the intricate epidemiology of *Campylobacter* cross-contamination in chicken slaughter and abattoir processing, offering detailed genetic insights into transmission dynamics and pinpointing potential contamination sources (Prendergast et al., 2022). Epidemiological investigations commonly employ SNP analysis, which compares isolates at the nucleotide level and offers high discriminatory power, to evaluate relatedness among isolates (Hasan et al., 2021; Foddai et al., 2022). Within the scope of this study, some clusters (displaying fewer than 20 SNP differences) were identified in pooled caecal samples, originating from both same-farm and different-farm flocks, and were also found on chicken carcasses from the respective flocks. Furthermore, genomic similarities between isolates from pooled ceca and those of carcasses across various processing stages and slaughter batches suggest that a considerable portion of carcass contamination may have originated within the slaughterhouse itself (Rossler et al., 2020; Prendergast et al., 2022).

Another notable finding in this study is the identification of shared clusters on carcasses from consecutively processed batches, indicating the transmission of strains between successive slaughter batches (Prendergast et al., 2022; Awad et al., 2023), previously documented but now illustrated for the first time in the UAE through genomic evidence. Nonetheless, some of this contamination may have arisen from fecal matter potentially contaminating external surfaces such as feathers during transportation from the farm to the slaughterhouse or slaughtering (Rasschaert et al., 2020). Additionally, *Campylobacter* contamination of crates could have further contaminated birds during transportation (Normand et al., 2008).

Interestingly, within one of the *C. jejuni* clusters (CJ2), we observed a relatedness between carcasses from flocks originating from both internal farms owned by the company and external farms contracted by the company. Upon further examination of the rearing conditions of these farms, it became apparent that they shared commonalities, such as the supply of one-day-old chicks, feed from the same mill, and supervision by the company’s veterinary team on externally contracted farms. This suggests a hypothesis that *Campylobacter* contamination in flocks from both internal and external farms may have originated from a shared source, such as the hatchery, feed mill, transportation trucks, or personnel (Normand et al., 2008). Some researchers have also suggested the potential for external sources of *Campylobacter* contamination, including hatcheries, breeder hens, or transportation (Normand et al., 2008; Rasschaert et al., 2020). Considering the hypothesized pathways of cross-contamination between internal and external farms, alongside the genomic evidence presented in this work, it is evident that effective *Campylobacter* management in broiler production and slaughterhouses requires a comprehensive, multi-faceted approach that addresses potential contamination sources at every stage of the poultry production and processing chain (Prendergast et al., 2022).

The genomic data provided further insights into resistance and virulence factors among a representative subset of *Campylobacter* isolates from various slaughter batches. Although most cases of campylobacteriosis are self-limiting, ciprofloxacin and erythromycin are recommended for empirical therapy in severe cases (Asuming-Bediako et al., 2019). The prevalence of the *gyrA* T86I mutation observed among these isolates aligns with previous reports, indicating its widespread occurrence as one of the primary mechanisms of quinolones (e.g., ciprofloxacin) resistance in *Campylobacter* strains from both animal and human sources (Nelson et al., 2007; Zhang et al., 2003). Alongside the *gyrA* mutation, an RNA mutation at 23S r.2075, associated with macrolide (e.g., erythromycin) resistance, was prominently detected in the characterized *C. coli* isolates (Figure 3). This specific point mutation is recognized as the predominant genetic determinant responsible for high levels of erythromycin resistance in *Campylobacter*, particularly prevalent in *C. coli* compared to *C. jejuni* (Vinueza-Burgos et al., 2017). The findings of this study are consistent with our previous observations in UAE retail chicken, highlighting concerns regarding the emergence of AMR genetic markers in *Campylobacter* from chicken, particularly against fluoroquinolones in *C. jejuni* and notably against erythromycin in *C. coli* (Habib et al., 2023). Multiple reports have indicated a high level of agreement (>95%) between resistance genotypes and phenotypes, particularly for (fluoro) quinolone and macrolide classes of antimicrobials (Zhao et al., 2015; Dahl et al., 2021; Habib et al., 2023). Therefore, adopting WGS-based approaches to identify genetic markers associated with phenotypic resistance to clinically significant antimicrobials should be strongly considered in national and regional antimicrobial monitoring programs for *Campylobacter* at the human-food-animal interface.

The WGS results revealed a diverse array of virulence factors, with certain factors prevalent across all isolates. These findings align with previous studies reporting the widespread presence of such genes in strains linked to food and clinical cases (Tegtmeyer et al., 2021; Prendergast et al., 2022). Conversely, specific virulence genes were notably more abundant (and, in some cases, exclusive) among *C. jejuni* isolates. For instance, essential flagellin structural genes governing motility and adhesion, such as *flaA* and *flaB*, were absent in all *C. coli* and a few *C. jejuni* isolates examined in this study. This echoes findings from a study in China utilizing WGS to characterize *Campylobacter* from chickens, indicating the scarcity of these genes in *C. coli* compared to their prevalence in *C. jejuni* isolates (Xiao et al., 2023). However, it is crucial to interpret these results cautiously, as they may not reflect species-specific features but instead could be associated with prevailing clonal groups (STs) in different settings. Additionally, we noted that certain known virulence factors were absent from all isolates, such as genes related to the type IV secretion system (*virB* gene cluster). In a previous study in the UAE, approximately half [53.3% (24/45)] of characterized *C. coli* genomes were found to harbor type IV secretion system genes, including *virB4, virB8, virB9, virB10, and virD4* (Habib et al., 2023). This study brings attention to the variability in the presence of some virulence factors among *Campylobacter* isolates, underscoring the need for further investigation into the factors influencing their distribution and potential implications for pathogenicity. *Campylobacter*-induced gastroenteritis is multifactorial, where sometimes the precise contributions of identified virulence genes to disease development remain incompletely understood (Prendergast et al., 2022).

While this study provides the first insights into *Campylobacter* dynamics and prevalence during abattoir processing in the UAE, several limitations warrant consideration and offer avenues for future research. Firstly, the study was conducted in a specific poultry establishment, potentially limiting the generalizability of findings to other facilities with differing processing practices and environmental conditions. Future studies are in progress to encompass a broader range of slaughterhouses to capture variability across the industry. Additionally, the study primarily focused on *Campylobacter* contamination rates and genomic characteristics, leaving room for further exploration of other potential contributors to contamination, such as biofilm formation or environmental reservoirs. Moreover, longitudinal studies tracking *Campylobacter* prevalence over time (e.g., consecutive farm production cycles) could offer a more comprehensive understanding of temporal trends and the efficacy of intervention strategies.

## 5 Conclusion

*Campylobacter* infection stands as a significant food safety and public health concern globally, and this study represents a crucial step toward addressing the dearth of data on *Campylobacter* dynamics in the UAE poultry industry. By examining contamination rates and genomic characteristics across slaughter line stages and batches, we shed light on the multifactorial nature of *Campylobacter* contamination and underscored the importance of a comprehensive approach to mitigate its spread. Furthermore, the identification of shared clusters between consecutive slaughter batches, alongside genomic evidence of cross-contamination, emphasizes the need for stringent hygiene practices and targeted interventions at every stage of broiler production and processing. Moving forward, continued microbiological surveillance efforts, coupled with advances in genomic technologies, will be essential in guiding effective *Campylobacter* management strategies to safeguard public health and ensure the safety of chicken meat products in the UAE and beyond. To manage antimicrobial use in poultry production and curb the spread of resistant *Campylobacter* strains, several strategies should be implemented. These include the reduction of prophylactic antibiotic use, promoting alternatives such as probiotics and prebiotics, and improving biosecurity measures. Additionally, policy recommendations for antimicrobial stewardship should encompass stricter regulations on antibiotic use, mandatory surveillance programs, and the promotion of responsible use practices among veterinarians and farmers. Implementing these strategies at both national and regional levels is crucial to mitigate the development and spread of antimicrobial resistance in *Campylobacter* and ensure the continued efficacy of existing antimicrobials.

## Data Availability

Whole genome sequencing data are available at the National Center for Biotechnology Information (BioProject no. PRJNA1112130).

## References

[ref1] Allen V. M. Bull S. A. Corry J. E. Domingue G. Jørgensen F. Frost J. A.. (2007). *Campylobacter* spp. contamination of chicken carcasses during processing in relation to flock colonisation. Int. J. Food Microbiol. 113, 54–61. doi: 10.1016/j.ijfoodmicro.2006.07.011, PMID: 17007949

[ref2] Asuming-Bediako N. Parry-Hanson Kunadu A. Abraham S. Habib I. (2019). *Campylobacter* at the human-food Interface: the African perspective. Pathogens 8:87. doi: 10.3390/pathogens8020087, PMID: 31242594 PMC6631673

[ref3] Awad A. Yeh H. Y. Ramadan H. Rothrock M. J. (2023). Genotypic characterization, antimicrobial susceptibility and virulence determinants of *Campylobacter jejuni* and *Campylobacter coli* isolated from pastured poultry farms. Front. Microbiol. 14:1271551. doi: 10.3389/fmicb.2023.1271551, PMID: 38029099 PMC10668334

[ref4] CDC (2019). Preliminary incidence and trends of infections with pathogens transmitted commonly through food — foodborne diseases active surveillance network, 10 U.S. sites, 2016–2019 MMWR Morb mortal. Available at: https://www.cdc.gov/foodnet/reports/prelim-data-intro-2019.html (Accessed March 30, 2024). 10.15585/mmwr.mm6917a1 PMC7206985 32352955

[ref5] Dahl L. G. Joensen K. G. Østerlund M. T. Kiil K. Nielsen E. M. (2021). Prediction of antimicrobial resistance in clinical *Campylobacter jejuni* isolates from whole-genome sequencing data. Eur. J. Clin. Microbiol. Infect. Dis. 40, 673–682. doi: 10.1007/s10096-020-04043-y, PMID: 32974772 PMC7979593

[ref6] Duarte A. S. Nauta M. J. (2015). Impact of microbial count distributions on human health risk estimates. Int. J. Food Microbiol. 195, 48–57. doi: 10.1016/j.ijfoodmicro.2014.11.024, PMID: 25506750

[ref7] EFSA (2020). Update and review of control options for *Campylobacter* in broilers at primary production. Available at: https://www.efsa.europa.eu/en/efsajournal/pub/6090 (Accessed March 20, 2024). 10.2903/j.efsa.2020.6090 PMC7448041 32874298

[ref8] Feldgarden M. Brover V. Gonzalez-Escalona N. Frye J. G. Haendiges J. Haft D. H.. (2021). AMRFinderPlus and the reference gene catalog facilitate examination of the genomic links among antimicrobial resistance, stress response, and virulence. Sci. Rep. 11:12728. doi: 10.1038/s41598-021-91456-0, PMID: 34135355 PMC8208984

[ref9] Foddai A. Takeuchi-Storm N. Høg B. B. Kjeldgaard J. S. Andersen J. K. Ellis-Iversen J. (2022). Assessing *Campylobacter* cross-contamination of Danish broiler flocks at slaughterhouses considering true flock prevalence estimates and ad-hoc sampling. Microb. Risk Anal. 21:100214. doi: 10.1016/j.mran.2022.100214

[ref10] Fourment M. Gibbs M. J. (2006). PATRISTIC: a program for calculating patristic distances and graphically comparing the components of genetic change. BMC Evol. Biol. 6:1. doi: 10.1186/1471-2148-6-1, PMID: 16388682 PMC1352388

[ref11] Habib I. Coles J. Fallows M. Goodchild S. (2019). A baseline quantitative survey of *Campylobacter* spp. on retail chicken portions and carcasses in metropolitan Perth, Western Australia. Foodborne Pathog. Dis. 16, 180–186. doi: 10.1089/fpd.2018.2554, PMID: 30457884

[ref12] Habib I. Ibrahim Mohamed M. Y. Ghazawi A. Lakshmi G. B. Khan M. Li D.. (2023). Genomic characterization of molecular markers associated with antimicrobial resistance and virulence of the prevalent *Campylobacter coli* isolated from retail chicken meat in the United Arab Emirates. Curr. Res. Food Sci. 6:100434. doi: 10.1016/j.crfs.2023.100434, PMID: 36687171 PMC9850066

[ref13] Habib I. Mohamed M. I. Khan M. (2021). Current state of *Salmonella*, *Campylobacter* and *Listeria* in the food chain across the Arab countries: a descriptive review. Foods 10:2369. doi: 10.3390/foods10102369, PMID: 34681418 PMC8535026

[ref14] Habib I. Mohamed M. Y. I. Lakshmi G. B. Khan M. Li D. (2022). Quantification of *Campylobacter* contamination on chicken carcasses sold in retail markets in the United Arab Emirates. Food Contaminat. 9:95. doi: 10.1186/s40550-022-00095-4

[ref15] Hakeem M. J. Lu X. (2021). Survival and control of *Campylobacter* in poultry production environment. Front. Cell. Infect. Microbiol. 10:615049. doi: 10.3389/fcimb.2020.615049, PMID: 33585282 PMC7879573

[ref16] Hansson I. Sandberg M. Habib I. Lowman R. Engvall E. O. (2018). Knowledge gaps in control of Campylobacter for prevention of campylobacteriosis. Transbound. Emerg. Dis. 65, 30–48. doi: 10.1111/tbed.12870, PMID: 29663680

[ref17] Hasan N. A. Davidson R. M. Epperson L. E. Kammlade S. M. Beagle S. Levin A. R.. (2021). Population genomics and inference of *Mycobacterium avium* complex clusters in cystic fibrosis care centers, United States. Emerg. Infect. Dis. 27, 2836–2846. doi: 10.3201/eid2711.210124, PMID: 34670648 PMC8544995

[ref18] ISO (2017). Microbiology of food and animal feeding stuffs – horizontal method for detection and enumeration of Campylobacter spp. – part 1: detection method (ISO 10272-1, 2006). Available at: https://www.iso.org/standard/63228.html (Accessed May 5, 2023).

[ref19] Keener K. M. Bashor M. P. Curtis P. A. Sheldon B. W. Kathariou S. (2004). Comprehensive review of *Campylobacter* and poultry processing. Compr. Rev. Food Sci. Food Saf. 3, 105–116. doi: 10.1111/j.1541-4337.2004.tb00060.x 33430546

[ref20] Lassen B. Takeuchi-Storm N. Henri C. Hald T. Sandberg M. Ellis-Iversen J. (2023). Analysis of reservoir sources of *Campylobacter* isolates to free-range broilers in Denmark. Poult. Sci. 102:103025. doi: 10.1016/j.psj.2023.103025, PMID: 37672837 PMC10485630

[ref21] Leone C. Xu X. Mishra A. Thippareddi H. Singh M. (2024). Interventions to reduce Salmonella and *Campylobacter* during chilling and post-chilling stages of poultry processing: a systematic review and meta-analysis. Poult. Sci. 103:103492. doi: 10.1016/j.psj.2024.103492, PMID: 38335673 PMC10864810

[ref22] Moilanen T. Lehtinen J. Visuri K. Sihvonen S. (2024). Solu – a cloud platform for real-time genomic pathogen surveillance. BioRxiv. doi: 10.1101/2024.05.30.596434 PMC11731562 39806295

[ref23] Nagel G. M. Bauermeister L. J. Bratcher C. L. Singh M. McKee S. R. (2013). *Salmonella* and *Campylobacter* reduction and quality characteristics of poultry carcasses treated with various antimicrobials in a post-chill immersion tank. Int. J. Food Microbiol. 165, 281–286. doi: 10.1016/j.ijfoodmicro.2013.05.016, PMID: 23800739

[ref24] Nelson J. M. Chiller T. M. Powers J. H. Angulo F. J. (2007). Fluoroquinolone-resistant *Campylobacter* species and the withdrawal of fluoroquinolones from use in poultry: a public health success story. Clin. Infect. Dis. 44, 977–980. doi: 10.1086/512369, PMID: 17342653

[ref25] Normand V. Boulianne M. Quessy S. (2008). Evidence of cross-contamination by *Campylobacter* spp. of broiler carcasses using genetic characterization of isolates. Canad. J. Vet. Res. 72, 396–402. Available at: https://www.ncbi.nlm.nih.gov/pmc/articles/PMC2568043/19086371 PMC2568043

[ref26] Pacholewicz E. Dame-Korevaar A. van der Most M. Ellen H. Bokma M. H. Koene M. G. J. (2024). *Campylobacter* presence on Dutch broiler farms and associated risk factors. Poult. Sci. 103:103568. doi: 10.1016/j.psj.2024.103568, PMID: 38447312 PMC11067780

[ref27] Pacholewicz E. Swart A. Schipper M. Gortemaker B. G. Wagenaar J. A. Havelaar A. H.. (2015). A comparison of fluctuations of *Campylobacter* and *Escherichia coli* concentrations on broiler chicken carcasses during processing in two slaughterhouses. Int. J. Food Microbiol. 205, 119–127. doi: 10.1016/j.ijfoodmicro.2015.04.006, PMID: 25950748

[ref28] Prendergast D. M. Lynch H. Whyte P. Golden O. Murphy D. Gutierrez M.. (2022). Genomic diversity, virulence and source of *Campylobacter jejuni* contamination in Irish poultry slaughterhouses by whole genome sequencing. J. Appl. Microbiol. 133, 3150–3160. doi: 10.1111/jam.15753, PMID: 35993276 PMC9804324

[ref29] Rasschaert G. De Zutter L. Herman L. Heyndrickx M. (2020). *Campylobacter* contamination of broilers: the role of transport and slaughterhouse. Int. J. Food Microbiol. 322:108564. doi: 10.1016/j.ijfoodmicro.2020.108564, PMID: 32163798

[ref30] Robyn J. Rasschaert G. Pasmans F. Heyndrickx M. (2015). Thermotolerant *Campylobacter* during broiler rearing: risk factors and intervention. Compr. Rev. Food Sci. Food Saf. 14, 81–105. doi: 10.1111/1541-4337.12124, PMID: 33401809

[ref31] Rosenquist H. Boysen L. Krogh A. L. Jensen A. N. Nauta M. (2013). *Campylobacter* contamination and the relative risk of illness from organic broiler meat in comparison with conventional broiler meat. Int. J. Food Microbiol. 162, 226–230. doi: 10.1016/j.ijfoodmicro.2013.01.022 23454812

[ref32] Rossler E. Olivero C. Soto L. P. Frizzo L. S. Zimmermann J. Rosmini M. R.. (2020). Prevalence, genotypic diversity and detection of virulence genes in thermotolerant *Campylobacter* at different stages of the poultry meat supply chain. Int. J. Food Microbiol. 326:108641. doi: 10.1016/j.ijfoodmicro.2020.108641, PMID: 32371295

[ref33] Seliwiorstow T. Baré J. Van Damme I. Uyttendaele M. De Zutter L. (2015). *Campylobacter* carcass contamination throughout the slaughter process of *Campylobacter*-positive broiler batches. Int. J. Food Microbiol. 194, 25–31. doi: 10.1016/j.ijfoodmicro.2014.11.004, PMID: 25461605

[ref34] Tegtmeyer N. Sharafutdinov I. Harrer A. Soltan Esmaeili D. Linz B. Backert S. (2021). *Campylobacter* virulence factors and molecular host-pathogen interactions. Curr. Top. Microbiol. Immunol. 431, 169–202. doi: 10.1007/978-3-030-65481-8_7, PMID: 33620652

[ref35] USDA, (2021). Poultry and products annual. Available at: https://www.fas.usda.gov/data/united-arab-emirates-poultry-and-products-annual-2. (Accessed January 12, 2022).

[ref36] Vinueza-Burgos C. Cevallos M. Cisneros M. Van Damme I. De Zutter L. (2018). Quantification of the *Campylobacter* contamination on broiler carcasses during the slaughter of *Campylobacter* positive flocks in semi-industrialized slaughterhouses. Int. J. Food Microbiol. 269, 75–79. doi: 10.1016/j.ijfoodmicro.2018.01.021, PMID: 29421361

[ref37] Vinueza-Burgos C. Wautier M. Martiny D. Cisneros M. Van Damme I. De Zutter L. (2017). Prevalence, antimicrobial resistance and genetic diversity of *Campylobacter coli* and *Campylobacter jejuni* in Ecuadorian broilers at slaughter age. Poult. Sci. 96, 2366–2374. doi: 10.3382/ps/pew487, PMID: 28339716 PMC5850218

[ref38] Xiao J. Cheng Y. Zhang W. Lu Q. Guo Y. Hu Q.. (2023). Genetic characteristics, antimicrobial susceptibility, and virulence genes distribution of *Campylobacter* isolated from local dual-purpose chickens in Central China. Front. Cell. Infect. Microbiol. 13:1236777. doi: 10.3389/fcimb.2023.1236777, PMID: 37743858 PMC10517862

[ref39] Zhang Q. Lin J. Pereira S. (2003). Fluoroquinolone-resistant *Campylobacter* in animal reservoirs: dynamics of development, resistance mechanisms and ecological fitness. Anim. Health Res. Rev. 4, 63–71. doi: 10.1079/ahr200356, PMID: 15134291

[ref40] Zhang X. Tang M. Zhou Q. Zhang J. Yang X. Gao Y. (2018). Prevalence and characteristics of *Campylobacter* throughout the slaughter process of different broiler batches. Front. Microbiol. 9:2092. doi: 10.3389/fmicb.2018.02092, PMID: 30233556 PMC6131577

[ref41] Zhao S. Tyson G. H. Chen Y. Li C. Mukherjee S. Young S.. (2015). Whole-genome sequencing analysis accurately predicts antimicrobial resistance phenotypes in *Campylobacter* spp. Appl. Environ. Microbiol. 82, 459–466. doi: 10.1128/AEM.02873-15, PMID: 26519386 PMC4711122

