# Supplementary material for: Quantitative assessment and genomic profiling of Campylobacter dynamics in poultry processing: a case study in the United Arab Emirates integrated abattoir system
Source: Front Microbiol. 2024 Sep 4;15:1439424. doi: 10.3389/fmicb.2024.1439424 (PMC11408311; doi:10.3389/fmicb.2024.1439424)
Supplement: Supplementary file 1 [file Data_Sheet_1.PDF]

Supplementary material for:

Quantitative Assessment and Genomic Profiling of *Campylobacter* Dynamics in Poultry Processing: A Case Study in the United Arab Emirates Integrated Abattoir System

Ihab Habib[1,2*, †], Mohamed-Yousif Ibrahim Mohamed[1, †], Glindya Bhagya Lakshmi[1], Hassan Mohamed Al Marzooqi[3], Hanan Sobhy Afifi[3], Mohamed Gamal Shehata[3,4], Mushtaq Khan[5], Akela Ghazawi[5], Afra Abdalla[1], Febin Anes[1]

[1] Veterinary Public Health Research Laboratory, Department of Veterinary Medicine, College of Agriculture and Veterinary Medicine, United Arab Emirates University, Al Ain, United Arab Emirates

[2] ASPIRE Research Institute for Food Security in the Drylands (ARIFSID), United Arab Emirates University, Al Ain, United Arab Emirates

[3] Food Research Section, Applied Research and Capacity Building Division, Abu Dhabi Agriculture and Food Safety Authority (ADAFSA), Abu Dhabi, United Arab Emirates

[4] Food Technology Department, Arid Lands Cultivation Research Institute (ALCRI), City of Scientific Research and Technological Applications (SRTACITY), Alexandria, Egypt

[5] Department of Medical Microbiology and Immunology, College of Medicine and Health Sciences, United Arab Emirates University, Al Ain, United Arab Emirates

*** Correspondence:**
Ihab Habib
i.habib@uaeu.ac.ae

Table S1 Single nucleotide polymorphism (SNP) distance matrix among *Campylobacter coli* isolates recovered from chicken carcasses sampled from different farms and abattoir processing stages (light red fill indicates less than 20 SNPs distance).

Campylobacter coli isolates	ADF_51	ADF_50	ADF_48	ADF_46	ADF_45	ADF_41	ADF_40	ADF_37	ADF_36	ADF_34	ADF_33	ADF_30	ADF_29	ADF_28	ADF_27	ADF_26	ADF_24	ADF_23	ADF_22	ADF_21	ADF_20	ADF_19	ADF_16	ADF_15	ADF_14	ADF_13	ADF_10	ADF_9	ADF_8	ADF_7	ADF_6	ADF_4
ADF_51	0	2760	1	1	2762	1910	1911	1911	1910	1910	2104	2104	2104	2103	2103	2103	2103	2104	2104	2103	1033	1874	1481	1482	1773	1481	2	2	1	1	2	1
ADF_50	2760	0	3077	3077	2	3442	3442	3442	3443	3443	3201	3201	3201	3202	3200	3202	3202	3201	3201	3202	2677	3421	3213	3210	3319	3211	3076	3076	3077	3077	3076	3077
ADF_48	1	3077	0	0	3079	2150	2151	2151	2152	2152	2439	2439	2439	2440	2438	2440	2440	2439	2439	2440	1193	2109	1677	1676	1993	1677	1	1	2	2	1	2
ADF_46	1	3077	0	0	3079	2150	2151	2151	2152	2152	2439	2439	2439	2440	2438	2440	2440	2439	2439	2440	1193	2109	1677	1676	1993	1677	1	1	2	2	1	2
ADF_45	2762	2	3079	3079	0	3444	3444	3444	3445	3445	3203	3203	3203	3204	3202	3204	3204	3203	3203	3204	2679	3423	3215	3212	3321	3213	3078	3078	3079	3079	3078	3079
ADF_41	1910	3442	2150	2150	3444	0	0	0	1	1	2681	2681	2681	2683	2681	2682	2682	2681	2681	2682	52	73	1753	1750	79	1751	2149	2149	2150	2150	2149	2150
ADF_40	1911	3442	2151	2151	3444	0	0	0	1	1	2682	2682	2682	2684	2682	2683	2683	2682	2682	2683	52	73	1753	1750	79	1751	2150	2150	2151	2151	2150	2151
ADF_37	1911	3442	2151	2151	3444	0	0	0	1	1	2682	2682	2682	2684	2682	2683	2683	2682	2682	2683	52	73	1753	1750	79	1751	2150	2150	2151	2151	2150	2151
ADF_36	1910	3443	2152	2152	3445	1	1	1	0	0	2683	2683	2683	2683	2683	2682	2682	2683	2683	2682	51	74	1752	1751	80	1750	2151	2151	2150	2150	2151	2150
ADF_34	1910	3443	2152	2152	3445	1	1	1	0	0	2683	2683	2683	2683	2683	2682	2682	2683	2683	2682	51	74	1752	1751	80	1750	2151	2151	2150	2150	2151	2150
ADF_33	2104	3201	2439	2439	3203	2681	2682	2682	2683	2683	0	0	0	2	0	1	1	0	0	1	1854	2648	2507	2506	2551	2507	2438	2438	2439	2439	2438	2438
ADF_30	2104	3201	2439	2439	3203	2681	2682	2682	2683	2683	0	0	0	2	0	1	1	0	0	1	1854	2648	2507	2506	2551	2507	2438	2438	2439	2439	2438	2438
ADF_29	2104	3201	2439	2439	3203	2681	2682	2682	2683	2683	0	0	0	2	0	1	1	0	0	1	1854	2648	2507	2506	2551	2507	2438	2438	2439	2439	2438	2438
ADF_28	2103	3202	2440	2440	3204	2683	2684	2684	2683	2683	2	2	2	0	2	1	1	2	2	1	1854	2650	2507	2508	2553	2507	2439	2439	2438	2438	2439	2437
ADF_27	2103	3200	2438	2438	3202	2681	2682	2682	2683	2683	0	0	0	2	0	1	1	0	0	1	1854	2648	2507	2506	2551	2507	2437	2437	2438	2438	2437	2437
ADF_26	2103	3202	2440	2440	3204	2682	2683	2683	2682	2682	1	1	1	1	1	0	0	1	1	0	1853	2649	2506	2507	2552	2506	2439	2439	2438	2438	2439	2437
ADF_24	2103	3202	2440	2440	3204	2682	2683	2683	2682	2682	1	1	1	1	1	0	0	1	1	0	1853	2649	2506	2507	2552	2506	2439	2439	2438	2438	2439	2437
ADF_23	2104	3201	2439	2439	3203	2681	2682	2682	2683	2683	0	0	0	2	0	1	1	0	0	1	1854	2648	2507	2506	2551	2507	2438	2438	2439	2439	2438	2438
ADF_22	2104	3201	2439	2439	3203	2681	2682	2682	2683	2683	0	0	0	2	0	1	1	0	0	1	1854	2648	2507	2506	2551	2507	2438	2438	2439	2439	2438	2438
ADF_21	2103	3202	2440	2440	3204	2682	2683	2683	2682	2682	1	1	1	1	1	0	0	1	1	0	1853	2649	2506	2507	2552	2506	2439	2439	2438	2438	2439	2437
ADF_20	1033	2677	1193	1193	2679	52	52	52	51	51	1854	1854	1854	1854	1854	1853	1853	1854	1854	1853	0	4	211	210	4	209	1192	1192	1191	1191	1192	1191
ADF_19	1874	3421	2109	2109	3423	73	73	73	74	74	2648	2648	2648	2650	2648	2649	2649	2648	2648	2649	4	0	1742	1739	0	1740	2108	2108	2109	2109	2108	2109
ADF_16	1481	3213	1677	1677	3215	1753	1753	1753	1752	1752	2507	2507	2507	2507	2507	2506	2506	2507	2507	2506	211	1742	0	1	1507	0	1676	1676	1675	1675	1676	1675
ADF_15	1482	3210	1676	1676	3212	1750	1750	1750	1751	1751	2506	2506	2506	2508	2506	2507	2507	2506	2506	2507	210	1739	1	0	1504	1	1675	1675	1676	1676	1675	1676
ADF_14	1773	3319	1993	1993	3321	79	79	79	80	80	2551	2551	2551	2553	2551	2552	2552	2551	2551	2552	4	0	1507	1504	0	1505	1992	1992	1993	1993	1992	1993
ADF_13	1481	3211	1677	1677	3213	1751	1751	1751	1750	1750	2507	2507	2507	2507	2507	2506	2506	2507	2507	2506	209	1740	0	1	1505	0	1676	1676	1675	1675	1676	1675
ADF_10	2	3076	1	1	3078	2149	2150	2150	2151	2151	2438	2438	2438	2439	2437	2439	2439	2438	2438	2439	1192	2108	1676	1675	1992	1676	0	0	1	1	0	1
ADF_9	2	3076	1	1	3078	2149	2150	2150	2151	2151	2438	2438	2438	2439	2437	2439	2439	2438	2438	2439	1192	2108	1676	1675	1992	1676	0	0	1	1	0	1
ADF_8	1	3077	2	2	3079	2150	2151	2151	2150	2150	2439	2439	2439	2438	2438	2438	2438	2439	2439	2438	1191	2109	1675	1676	1993	1675	1	1	0	0	1	0
ADF_7	1	3077	2	2	3079	2150	2151	2151	2150	2150	2439	2439	2439	2438	2438	2438	2438	2439	2439	2438	1191	2109	1675	1676	1993	1675	1	1	0	0	1	0
ADF_6	2	3076	1	1	3078	2149	2150	2150	2151	2151	2438	2438	2438	2439	2437	2439	2439	2438	2438	2439	1192	2108	1676	1675	1992	1676	0	0	1	1	0	1
ADF_4	1	3077	2	2	3079	2150	2151	2151	2150	2150	2438	2438	2438	2437	2437	2437	2437	2438	2438	2437	1191	2109	1675	1676	1993	1675	1	1	0	0	1	0

Table S2 Single nucleotide polymorphism (SNP) distance matrix among *Campylobacter jejuni* isolates recovered from chicken carcasses sampled from different farms and abattoir processing stages (light red fill indicates less than 20 SNPs distance).

Campylobacter jejuni isolates	ADF_49	ADF_47	ADF_44	ADF_42	ADF_38	ADF_35	ADF_32	ADF_31	ADF_25	ADF_18	ADF_17	ADF_11	ADF_5	ADF_3	ADF_2	ADF_1
ADF_49	0	2	1	6614	6611	4502	6600	6601	6618	4500	4500	6612	6612	6611	4502	4500
ADF_47	2	0	3	6614	6613	4502	6600	6601	6618	4500	4502	6612	6614	6613	4502	4500
ADF_44	1	3	0	6613	6610	4501	6601	6600	6617	4501	4501	6611	6611	6610	4501	4501
ADF_42	6614	6614	6613	0	7	6693	31	30	12	6693	6694	4	6	5	6694	6694
ADF_38	6611	6613	6610	7	0	6690	32	31	13	6690	6689	5	5	4	6691	6691
ADF_35	4502	4502	4501	6693	6690	0	6678	6677	6695	1	2	6691	6693	6692	2	3
ADF_32	6600	6600	6601	31	32	6678	0	1	30	6676	6677	29	31	30	6679	6677
ADF_31	6601	6601	6600	30	31	6677	1	0	29	6677	6678	28	30	29	6678	6678
ADF_25	6618	6618	6617	12	13	6695	30	29	0	6695	6696	10	12	11	6696	6696
ADF_18	4500	4500	4501	6693	6690	1	6676	6677	6695	0	1	6691	6693	6692	3	2
ADF_17	4500	4502	4501	6694	6689	2	6677	6678	6696	1	0	6692	6692	6691	4	3
ADF_11	6612	6612	6611	4	5	6691	29	28	10	6691	6692	0	2	1	6692	6692
ADF_5	6612	6614	6611	6	5	6693	31	30	12	6693	6692	2	0	1	6694	6694
ADF_3	6611	6613	6610	5	4	6692	30	29	11	6692	6691	1	1	0	6693	6693
ADF_2	4502	4502	4501	6694	6691	2	6679	6678	6696	3	4	6692	6694	6693	0	1
ADF_1	4500	4500	4501	6694	6691	3	6677	6678	6696	2	3	6692	6694	6693	1	0
